# Equity in Health Care: A Qualitative Study with Refugees, Health Care Professionals, and Administrators in One Region in Germany

**DOI:** 10.1155/2020/4647389

**Published:** 2020-02-24

**Authors:** Karolin Hahn, Jost Steinhäuser, Katja Goetz

**Affiliations:** Institute of Family Medicine, University Hospital Schleswig-Holstein, Campus Luebeck, Ratzeburger Allee 160, 23538 Luebeck, Germany

## Abstract

*Introduction*. Equity in health is an essential issue and it would appear that it is not guaranteed for all human beings, especially refugee groups. The aim of this qualitative descriptive study was to explore the experiences of refugees, health care professionals, and administrators of refugee health care in a host country.

**Methods:**

The study used qualitative methods which consisted of a convenience sample of stakeholders directly and indirectly involved in care for refugees and refugees themselves. The study participants were located in a rural area in the federal state of Schleswig-Holstein, Germany. Focus groups and interviews were conducted with 25 participants. A semistructured interview guideline was used for the focus groups and interviews. The data were evaluated using qualitative content analysis.

**Results:**

Four main categories were identified which are important for equity in health care: legal aspects, sociocultural aspects, environmental aspects, and communication aspects. Legal frameworks and language barriers were perceived as strong barriers for accessing health care.

**Conclusions:**

The findings suggest that the host countries should address the specific needs of this population group at a systemic and individual level. Based on the views of the participants interviewed it can be concluded that the refugee population group is particularly affected by limited access to health care services. Bureaucratic barriers, unfamiliarity with a new health system, and language issues all contribute to limiting access to health care services.

## 1. Introduction

“Equity in health implies that ideally everyone should have a fair opportunity to attain their full health potential and, more pragmatically, that no one should be disadvantaged from achieving this potential if it can be avoided” [1]. The opportunity to obtain equity in health across a nation can be influenced by structural (e.g., health system organisation), contextual (e.g., access to health care providers), and individual (e.g., linguistic aspects) factors which determine adequate access to health care [2]. There are many innovations and projects which aim to reduce inequalities in health and to promote equity in health, such as the European portal for action on health inequalities [3] or the German cooperation network “Equity in Health” [4]. Different reviews showed that migrant populations in particular suffer disadvantages in accessing health care [2, 5]. A lower utilization of specialist care, the use of medication, and therapist consultation were found amongst migrant populations [6].

The special challenge for health care in this population group was also recognised by the World Organisation of Family Doctors (WONCA) which is reflected in the following statement “Refugees should have access to equitable, affordable and high-quality health care services in all Europe” [7]. In recent years, an increasing number of refugees were registered in different European countries. By the end of 2016, there were approximately 5.2 million refugees in Europe, and 669,500 of these were in Germany [8]. This fact results in challenges for health care delivery and health care providers such as access to health care and in dealing with cultural differences and language barriers [5, 9]. In Germany, the Asylum Seekers Benefits Act regulates health care for refugees and asylum seekers. According to German law, only treatment for acute illnesses and pain is covered [10, 11]. The European Social Charter is a Council of Europe treaty and Article 11 expresses the right to the highest attainable standards of health for European citizens [12].

However, for different stakeholders the question remains how equity in health care can be guaranteed. Different studies show that, for example, the implementation of an electronic health card or patient-held health records could influence equity in refugee health care positively [13]. These different implementation strategies could have a positive impact on the continuity of care and facilitate access to primary care that is important for reducing inequities [13–15]. For an explanation of equity in health, different factors should be considered such as biological, sociopolitical, and environmental factors [16]. For research on refugee health care with a focus on equity in health, these different factors play an essential role in understanding potential health care gaps.

The term “refugee” must be distinguished from “migrant.” Refugees are persons who are forced to leave their country of origin due to persecution, war, or generalized violence. They require international protection. Migrants are persons who decide to move away from their country of origin without regard for their legal status and for a variety of reasons for migration [17].

Little is known about the perception of health care by refugees in Germany. Therefore, the aim of this qualitative descriptive study was to explore the experiences of refugees, health care professionals, and administrators involved in refugee health care in Germany.

## 2. Materials and Methods

### 2.1. Study Design

The present study used qualitative methods. Focus groups and interviews were held using best practice guidelines for qualitative studies [18]. In addition to the refugee perspective and in order to receive comprehensive insights into the medical care of refugees, we used a convenience sample of stakeholders directly and indirectly involved in refugee care. The stakeholders directly involved included health care professionals (physicians and practice assistants). Stakeholders involved indirectly included administrators responsible for refugee matters. “Indirectly” refers to people who do not provide patient care but are responsible for administration, such as employees from the social welfare office. The qualitative study design was chosen to allow an intensive analysis of motives, attitudes, and needs of participants. A semistructured interview guideline was used for the focus groups and interviews.

### 2.2. Participants and Recruitment

The participated refugees were individuals living in refugee accommodation in rural areas. The interviewed health care professionals and administrators were located in Schleswig-Holstein. Refugee accommodation consists of special housing where refugees are required to live. A qualitative study describing the living conditions in such refugee accommodation in the South of Germany was recently published [19].

In order to recruit refugees for the study, the facility manager of the refugee accommodation informed the refugees about the study with the help of interpreters. The interpreter provided spoken and written information. The facility manager informed us about the refugees who were interested in participating in the study. The facility manager communicated two potential appointments to the refugees who then chose one of the appointments and relayed their choice via the facility manager. The inclusion criteria for refugees in this study was a minimum age of 18 and at least one visit to outpatient care within the last 6 months since their arrival in Germany. The health care professionals were responsible for health care in refugee accommodation as part of a project financed by the Damp Siftung called “Mobile Medical Practice” (Rollende Arztpraxis). In this practice, the health care professionals provide general practice care for refugees in a rural region of Schleswig-Holstein and were asked to participate in this study [20]. Administrators were recruited by the Migration and Health Working Group Schleswig-Holstein. This working group consists of over 40 people who meet four times a year to discuss refugee health care in Schleswig-Holstein. KH and KG regularly participated in the working group and gave a short speech about the project during the meeting. At the end of the meeting, participants were given the chance to indicate their interest in participating. Contact details were exchanged between KH, KG, and interested parties.

Recruitment to the focus groups and interviews took place between July 2016 and February 2017. KH managed the health care professionals and potentially interested parties from the working group mentioned. Together they chose a mutually acceptable appointment time. KH carried out the interviews and focus group with health care professionals and administrators. A German-speaking researcher conducted the focus groups with the refugees with the help of trained interpreters, who asked participants questions on behalf of the researcher. The interpreters translated the refugees' responses back into German during the focus group discussion. Refugees were only included in focus groups whereas health care professionals participated in the focus group and the interviews and administrators only in the interviews. This resulted in a combination of data from focus groups and individual interviews. This combination had the potential for identification of individual and contextual circumstances and enhanced data richness [21]. Participation in the focus groups or interviews was voluntary. Study participants signed an informed consent form in advance of participation. After content saturation was reached, no further efforts for recruitment were undertaken. According to the literature as criteria for content saturation the researcher team decided that whether no new themes and no new coding were examined the recruitment stopped [22, 23].

### 2.3. Data Collection and Analyses

An interview guide for the focus groups and interviews was developed by an interprofessional team of physicians, sociologists, health service researchers, and health scientists. The interview guide focused on the current refugee health care situation and experiences of refugees with health care. A team consisting of a social scientist and health care professionals developed the interview guide based on a systematic review, personal experiences, and the aims of the study [24]. For refugees, the interview guide for the focus groups included the following questions:

1. What is your experience of medical care to date?
2. What do you think about your medical care at the moment?
3. What should your medical care look like in the future?
4. For health care professionals and administrators the interview guide for the focus group and interviews included the following questions:
5. How do you perceive the medical care of refugees at the moment?
6. Which are the needs of refugee medical care?
7. How should the medical care of refugees be organised in the future?

Data collection was carried out from August 2016 to March 2017. The focus groups and interviews were digitally recorded. The collected data were transcribed in full and anonymized. All transcripts were in German and their accuracy was validated by comparing the transcripts with the digital recorded focus groups and interviews. A qualitative content analysis was then carried out on the transcripts [25, 26]. This is a well-known approach in health care science and often used for interpreting text material [27]. The structured approach of content analysis allows a descriptive view of the health care situation of refugees from different perspectives. The analysis of the data used an iterative approach. Specifically, the researcher used a deductive-inductive approach in generating thematic categories. Based on the interview guide, a provisional category system was created initially consisting of the current health care situation and health care needs (deductive approach). In the course of the analysis this was adapted according to the content of the transcripts and supplemented by emerging new categories (inductive approach) [25]. Each transcript was coded into main categories and subcategories independently by two researchers (KH, KG) and discussed in consensus meetings with a third researcher (JS). Quotes were used to illustrate each of the categories [25]. The same approach to analysis was carried out for both interviews and the focus groups. Specific illustrative quotes were translated into English. Together with detailed documentation of the research process, the quality principle of intersubjectivity and transparency was achieved [28]. These criteria are important to enhance the quality of qualitative studies and are responsible for understanding the research process as well as the interpretation and reporting of the results [29].

### 2.4. Ethical Approval and Consent to Participate

Ethical approval for this research was obtained from the Ethics Committee at the University of Luebeck in March 2016 (Approval No. 16-041). An informed consent form was signed by the participants for participation in the study.

## 3. Results

### 3.1. Sample Characteristics

A total of 25 participants were included in the focus groups and interviews. Two focus groups were held with refugees (*n* = 12) and one focus group was held with health professionals (*n* = 3). The refugees interviewed had been in Germany between 6 and 19 months (mean = 10.5 months). The focus groups lasted between 28 and 57 minutes (mean = 43 minutes). In addition, 10 interviews were carried out, 4 with health care professionals and 6 with administrators. The interviews lasted between 10 and 47 minutes (mean = 24 minutes). Most of the participants were male (72%, *n* = 18) and the mean age was 42. All of the interviewed health care professionals had many years of experience in general practice care and had been actively involved in refugee health care since 2015. A detailed description of the study participants is given in Table 1.

### 3.2. Main Categories

Four main categories were identified for describing equity in health care. These were legal aspects, sociocultural aspects, environmental aspects, and communication aspects. These main categories were divided into different subcategories and an overview is presented in Table 2.

### 3.3. Legal Aspects

The legal aspects related to the law, the Asylum Seekers Benefits Act. and the involvement of government agencies. The Asylum Seekers Benefits Act was mentioned as a legal framework which covered asylum seekers in their first months in Germany and resulted in limited access to the social security system. An administrator stated:

“We have the Asylum Seekers Benefits Act, many of the clients are in the middle of the asylum process, they are entitled under the Asylum Seekers Benefits Act and that is very limited, especially in the first 15 months there is nothing, but also everything that comes afterwards is rather difficult.” (I_V1)

Moreover, interviewed health professionals perceived this legal framework as a barrier for accessing health care as the following statement illustrates:

“And of course this makes it very difficult in everyday life, so if a doctor first has to think about what the Government agencies will approve and what they will not approve, then, yes, it can be difficult for the medical side of the process.” (I_G1)

The work of government agencies was criticised in the participants' statements. One person highlighted the government agencies' inconsistency in decision-making as follows:

“… one case you do not succeed, another case everything goes smoothly, so it's all rather inconsistent you could say.” (I_G1)

One refugee reported his own experience with the government agencies. The interpreter translated the statement of the refugee as follows:

“He says it's a bit difficult, because everything has to be approved first, then you go through social services, then the district, then, for example, a health insurer, then the hospital; and he says it takes time to get anything done eventually.” (FG_1_T5)

The administrators reported that government agency employees often try to keep things running smoothly. However, established structures and legal requirements are obstacles to simple solutions.

*“Oh, very different from district to district. I would say that everyone involved is looking for the quickest solutions. But sometimes it's not possible because the existing structures are very bureaucratic. The bureaucratic hurdles are still very* high” (I_V2).

### 3.4. Sociocultural Aspects

The main category “sociocultural aspects” can be clarified with the subcategories “intercultural openness,” “general cultural aspects,” and “familiarity with the health care system.” “Intercultural openness refers to an openness to other cultures. In this context the administrators and health care professionals stated in particular that health services for refugees should be more open to intercultural exchange which would allow for cultural differences to be addressed. The following statement from an administrator emphasised this point:

“*Open up to the target group too. Open up to seeing that they come from a different cultural context and have different attitudes towards physicians.” (I_V2)*

Some of the interviewed refugees expressed a desire for an end to being disadvantaged and that they would prefer to be treated equally. The following statement highlights this request: *“We are all human beings and the physician should treat all equally, irrespective of their place of origin.” (FG_1_T2*).

Health care providers noticed another barrier in cultural differences in refugee health care related to the gender of health care professionals.

*“Like men who are not happy with urology in the first place, especially when the urologist is female. It's* impossible *to explain to my [ethnicity] patients that they have to visit a female urologist. That's really a problem, that's a specific problem.” (FG_G1_T1)*

A further barrier to health care access was a lack of familiarity with the health care system. Mastering the bureaucratic barriers without German language skills constitutes a great and often insurmountable barrier. One administrator stated:

“So refugees […] cannot do it all on their own, these bureaucratic barriers are very difficult to deal with for somebody who does not know the system.” (I_V2)

### 3.5. Environmental Aspects

Access to health care, mobility, availability of health care staff, and continuity of care were subcategories of the environmental aspects category. Access to health care for refugees is restricted in Germany by the Asylum Seekers Benefits Act. However, barriers to access do not only affect refugees. The administrators also had to deal with other barriers in refugee care, emphasised by the following statement:

“… what possibilities there are, that's not so easy to understand, like where to submit which application, how to get support with integration, who has access to medical care, how to organize...” (I_V4)

As the interviews and focus groups demonstrated, additional barriers existed in transport and the availability of health care staff in rural areas. Refugees stated that sometimes it was difficult to visit a physician, being limited by the lack of local public transport and health care staff in the area of their accommodation. They often had to resort to using the head of the accommodation as the driver to the nearest physician. The refugees within the focus group also stated that it was difficult to get an appointment with psychiatrists. The waiting times were too long. A further barrier for the refugees was obtaining the permission of the social welfare office for seeing a specialist. The administrators acknowledge the issue of transport, as illustrated by the following statement:

“Sometimes just the issue of driving in the rural areas. We have specialist doctors in <city>, but it's very hard to get there using public transport, and uh that's what we do then, we send them to these appointments.” (I_V3)

Continuity of care cannot be guaranteed due to the specific living conditions of refugees in the host country. The transition between hospital care and outpatient care also can lead to frustrations from the perspective of health care providers. They reported:

“But this can be quite difficult with the current setup. What currently does not happen is for the hospital to ask the patient where they will continue their treatment, to look up the contact in question and tells the patient that they're forwarding the relevant information for clarification, the way it happens with normal patients.” (FG_G1_T2)

### 3.6. Communication Aspects

Communication aspects in the form of language barriers and the availability of interpreters limit access to health care. Participants stated that the most important challenge in care and support of refugees was language. An administrator stated: “*Then there is the language barrier, that's a big problem, the social isolation.” (I_V1)* A health care professional also mentioned the difficulties in caring for patients in multiple languages:

“In my opinion the languages are also different. To say one thing, you need a lot more words in some languages and you even have to ask again, ask questions and stuff like that and then the answer is only “yes”.” (FG_G1_T1)

To overcome language barriers the best option was to work with professional interpreters. One health care professional emphasised the benefits of working with an interpreter

“And then you need an interpreter and then it works like with any other German patient. ”(FG_G1_T1)

One health care professional suggested that working with interpreters could be improved because of the additional time requirements when doing consultations with an interpreter and because interpreters were not available everywhere.

“And yes, there is a communication problem. Especially because the interpreters are not available nationwide and when you need them.” (I_G1)

Other than working with professional interpreters on-site, other possibilities were discussed to reduce languages barriers, such as a telephone interpretation service:

“Such a (…) phone number (…) that would be a great thing, if I had a phone number here that I could, maybe with a menu where I say yes, I need Arabic, I need Kurdish, I need Serbo-Croatian. And then with someone at the other end who translates for me on the phone.” (FG_G1_T2)

## 4. Discussion

The purpose of this study was to explore the experiences of refugees, health care professionals, and administrators in the health care system. The main findings of our study showed that legal aspects, sociocultural aspects, environmental aspects, and communication aspects are important for health care and that these factors have an impact on equity in health care.

Strong restrictions on health care access are due to legal aspects which can lead to helplessness for refugees but also for health care providers and administrators. To have access to health care dependant on one's residence status is a violation of the right to health but this structural barrier is conditioned by law and regulatory restrictions despite Article 11 of European Social Charter which sets out the right to protection of health for everyone [2, 10, 12]. Such legal restrictions can also lead to higher costs for the health care system [11]. The Report of the Migrant Integration Policy Index shows that there is still room for improvement in the field of health policy in different countries in the European Union but also in Germany [30]. An additional cost factor is the increased mental health burden within the refugee group [31, 32]. One possible strategy for reducing costs was shown by a modelling study in Germany which recommended screening for depression among refugees to identify patients in need of care [33].

A further barrier to ensure equity in health care is determined by sociocultural aspects. Different studies show that there is a need for a more open intercultural approach to meet the requirements of the different cultural backgrounds of the migrant population [34–36]. Cultural competence is often not part of the medical curriculum. The Lancet Commission on Culture and Health stated that the promotion and development of cultural competence should be an integral part of medical curricula [37]. Different recommendations to integrate cultural competence within medical education are given by Mews et al. [38]. Furthermore, WONCA recommended “appropriate training for GPs on cultural differences” [7].

Environmental aspects are an important factor in gaining access to health care and also depend on transport, availability of health care staff, and continuity of care. Different approaches are available which address barriers such as access to health care, transport, and availability of health care staff. Telemedicine could be a promising strategy [39]. It was found that e-health might be a facilitator in gaining access to health care as well as to overcoming transport barriers [40, 41]. It was also recommended that strengthening continuity of care for refugees could be achieved through patient-held personal health records [42].

As illustrated in our main findings, communication, especially the linguistic aspect, was one of the key barriers to equity in health care. Working with professional interpreters could be an important way of increasing equity in health care at the individual level. However, the use of professional interpreters brings with it its own set of challenges, such as the availability of trained interpreters, time issues, and also often translation difficulties (does the interpreter relay exactly what is intended?) [43, 44]. An alternative concept could be the use of telephone interpreting as mentioned by our participants. For example, Australian general practitioners have the option of using a free nationwide Telephone Interpreter Service [45]. However, if equity in health care is to be ensured, there is an urgent need for an adequate concept to address communication barriers in refugee health care. Another option might be to find physicians who are confident in a second language, a strategy which could also address the issue of dealing with patients from different cultural backgrounds [46].

The results reported from the focus groups and interviews with refugees and health care professionals are not specific to Germany. Our results are comparable to previous research with refugees and health professionals which observed that linguistic and cultural differences are the main barriers for refugees in accessing health care [45, 47, 48]. The interviewed administrators in our study complemented the perspective of health care professionals. Bureaucratic aspects and having to navigate a different health care system represent barriers to ensuring continuity of health care and were also observed outside Germany [43, 49].

As the results show, different gaps can be evaluated within the framework of equity in health. Finding solutions will depend on different conditions at the structural, organizational, and individual levels. The government should create conditions which enable everyone to exercise their right to health. Further studies should focus on how the existing gaps can be prevented, avoided, and justified [16]. Furthermore, barriers to accessing health care do not only affect refugees. The inequalities in health care utilization can be explained with the social model of health. Social, cultural, political, and economic factors influence individual health [50].

### 4.1. Strengths and Limitations

The findings of the current study must be viewed under the specific quality criteria for qualitative research. Some limitations have to be considered when interpreting the results. The study was undertaken in a rural area in one federal state in Germany and only included refugees, health care professionals, and administrators who were interested in taking part in this study, which may have resulted in selection bias. As usual in qualitative studies, the sample is not intended to be representative. No interrater reliability was analysed to examine the reliability of coding between the two researchers (KH, KG). Furthermore, we cannot exclude the possibility that results have been lost by working with interpreters in two out of three refugee focus groups. Conducting the focus groups with refugees was an organisational challenge and depended on factors such as time for participation in a group discussion, confidence in this specific situation, availability of interpreters, and economic aspects. The planned focus group with administrators did not take place due to a lack of interest in this target group. However, we were able to carry out personal interviews with administrators. Most of the interviewed refugees were male. The gender imbalance could have an impact on the interpretation of the findings. Only male refugees lived at one of the participating accommodation centres. Therefore, further qualitative research studies with refugees should organise separate male and female groups which in our case might have helped women to express their opinions more freely. We did not assess the educational qualifications of the refugees which may have influenced the statements in group discussion. Furthermore, we only involved refugees who lived in refugee accommodation. Further research is necessary to see whether the findings are also relevant for refugees who are not living in refugee accommodation. One further possible cause of bias could be that KG and KH were members of the Migration and Health Working Group Schleswig-Holstein because the administrators were recruited from this working group and were interviewed by KH.

The data support the importance of refugee health care with a specific focus on equity in health care and contributes to the development of hypotheses for further quantitative research. The results of this qualitative study cannot be generalized but are important for the generation of ideas and hypotheses as is the purpose of qualitative research in general.

## 5. Conclusions

Equity in health care should be an achievable goal for all human beings. Legal aspects, sociocultural aspects, environmental aspects, and communication aspects play an essential role in ensuring equity. Refugees as a population group are particularly affected by limited opportunities in accessing adequate health care. This access limitation can be magnified by bureaucratic aspects, having to navigate a different health care system but also by language barriers. Host countries are encouraged to address their specific needs at a systemic as well as individual level and a variety of social and institutional changes are required.

## Figures and Tables

**Table 1:** Overview of the conducted focus groups and interviews.

Participants	Interview type	Label	Number of participants (*n* = 25)	Gender of participants		Age, mean	Country of origin
				Male, *n*	Female, *n*		
Refugees	Focus groups	FG_1_TX or FG_2_TX	12	9	3	36.4	Iraq (*n* = 7),
							Afghanistan (*n* = 5)

Health care professionals	Focus group and interviews	FG_G1_TX or I_GX	7	6	1	48.2	—

Administrators	Interviews	I_VX	6	3	3	49.7	—

*n*: Number; FG: focus group; T: participant; X: number of participants; G: health care professional; I: interview; V: administrator.

**Table 2:** Main categories and subcategories of equity in health care.

Main categories	Subcategories
Legal aspects	-Law
	-Asylum seekers benefits act
	-Involvement of government agencies

Sociocultural aspects	-Intercultural openness
	-General cultural aspects
	-Familiarity with the health care system

Environmental aspects	-Access to health care
	-Mobility
	-Availability of health care staff
	-Continuity of care

Communication aspects	-Language barriers
	-Availability of interpreters

## Data Availability

The datasets used and analysed during the current study are available from the corresponding author upon reasonable request.

## Supplementary Materials

Supplementary Materials The SRQR checklist for qualitative research.
