# Supplementary material for: Equity in Health Care: A Qualitative Study with Refugees, Health Care Professionals, and Administrators in One Region in Germany
Source: Biomed Res Int. 2020 Feb 24;2020:4647389. doi: 10.1155/2020/4647389 (PMC7060884; doi:10.1155/2020/4647389)
Supplement: Supplementary Materials — The SRQR checklist for qualitative research. [file 4647389.f1.pdf]

Table 1

Standards for Reporting Qualitative Research (SRQR)[a]

No.	Topic	Item
	**Title and abstract**	
S1	Title page 1	Concise description of the nature and topic of the study Identifying the study as qualitative or indicating the approach (e.g., ethnography, grounded theory) or data collection methods (e.g., interview, focus group) is recommended
S2	Abstract page 2	Summary of key elements of the study using the abstract format of the intended publication; typically includes background, purpose, methods, results, and conclusions
	**Introduction**	
S3	Problem formulation page 3 - 4	Description and significance of the problem/phenomenon studied; review of relevant theory and empirical work; problem statement
S4	Purpose or research question page 4	Purpose of the study and specific objectives or questions
	**Methods**	
S5	Qualitative approach and research paradigm page 4, 7	Qualitative approach (e.g., ethnography, grounded theory, case study, phenomenology, narrative research) and guiding theory if appropriate; identifying the research paradigm (e.g., postpositivist, constructivist/ interpretivist) is also recommended; rationale[b]
S6	Researcher characteristics and reflexivity page 6	Researchers' characteristics that may influence the research, including personal attributes, qualifications/experience, relationship with participants, assumptions, and/or presuppositions; potential or actual interaction between researchers' characteristics and the research questions, approach, methods, results, and/or transferability
S7	Context page 5 & 6	Setting/site and salient contextual factors; rationale[b]
S8	Sampling strategy page 5	How and why research participants, documents, or events were selected; criteria for deciding when no further sampling was necessary (e.g., sampling saturation); rationale[b]
S9	Ethical issues pertaining to human subjects page 8	Documentation of approval by an appropriate ethics review board and participant consent, or explanation for lack thereof; other confidentiality and data security issues
S10	Data collection methods page 6 - 8	Types of data collected; details of data collection procedures including (as appropriate) start and stop dates of data collection and analysis, iterative process, triangulation of sources/methods, and modification of procedures in response to evolving study findings; rationale[b]
S11	Data collection instruments and technologies page 6 & 7	Description of instruments (e.g., interview guides, questionnaires) and devices (e.g., audio recorders) used for data collection; if/how the instrument(s) changed over the course of the study
S12	Units of study page 8	Number and relevant characteristics of participants, documents, or events included in the study; level of participation (could be reported in results)
S13	Data processing page 7	Methods for processing data prior to and during analysis, including transcription, data entry, data management and security, verification of data integrity, data coding, and anonymization/deidentification of excerpts
S14	Data analysis page 7 - 8	Process by which inferences, themes, etc., were identified and developed, including the researchers involved in data analysis; usually references a specific paradigm or approach; rationale[b]
S15	Techniques to enhance trustworthiness page 8	Techniques to enhance trustworthiness and credibility of data analysis (e.g., member checking, audit trail, triangulation); rationale[b]
	**Results/findings**	
S16	Synthesis and interpretation page 8 -15	Main findings (e.g., interpretations, inferences, and themes); might include development of a theory or model, or integration with prior research or theory
S17	Links to empirical data page 8-15	Evidence (e.g., quotes, field notes, text excerpts, photographs) to substantiate analytic findings
	**Discussion**	
S18	Integration with prior work, implications, transferability, and contribution(s) to the field page 16-18	Short summary of main findings; explanation of how findings and conclusions connect to, support, elaborate on, or challenge conclusions of earlier scholarship; discussion of scope of application/ generalizability; identification of unique contribution(s) to scholarship in a discipline or field
S19	Limitations page 18 - 19	Trustworthiness and limitations of findings

(*Table continues*)

Table 1
(Continued)

No.	Topic		Item
	**Other**		
S20	Conflicts of interest	page 18	Potential sources of influence or perceived influence on study conduct and conclusions; how these were managed
S21	Funding	page 18	Sources of funding and other support; role of funders in data collection, interpretation, and reporting
